# The Metabolic Syndrome and Mind-Body Therapies: A Systematic Review

**DOI:** 10.1155/2011/276419

**Published:** 2011-05-18

**Authors:** Joel G. Anderson, Ann Gill Taylor

**Affiliations:** Center for the Study of Complementary and Alternative Therapies, University of Virginia School of Nursing, P.O. Box 800782, Charlottesville, VA 22908-0782, USA

## Abstract

The metabolic syndrome, affecting a substantial and increasing percentage of the worldwide population, is comprised of a cluster of symptoms associated with increased risk of type 2 diabetes, cardiovascular disease, and other chronic conditions. Mind-body modalities based on Eastern philosophy, such as yoga, tai chi, qigong, and meditation, have become increasingly popular worldwide. These complementary therapies have many reported benefits for improving symptoms and physiological measures associated with the metabolic syndrome. However, clinical trial data concerning the effectiveness of these practices on the syndrome as a whole have not been evaluated using a systematic and synthesizing approach. A systematic review was conducted to critically evaluate the data from clinical trials examining the efficacy of mind-body therapies as supportive care modalities for management of the metabolic syndrome. Three clinical trials addressing the use of mind-body therapies for management of the metabolic syndrome were identified. Findings from the studies reviewed support the potential clinical effectiveness of mind-body practices in improving indices of the metabolic syndrome.

## 1. Introduction

The last 50 years have seen a dramatic increase in metabolic disorders, including obesity and type 2 diabetes, with the number of individuals diagnosed with type 2 diabetes worldwide expected to surpass 360 million by 2030 [1]. This prevalence is rising given the epidemic of obesity, which is fueled, in part, by physical inactivity and unhealthy eating patterns [2]. The metabolic syndrome, which affects nearly 40% of the US population [3], is a complex condition characterized by multiple, interrelated metabolic abnormalities linked to insulin resistance [4] and exacerbated by aging [5]. Core features of the metabolic syndrome are insulin resistance, glucose intolerance, atherogenic dyslipidemia, visceral adiposity, and hypertension [6, 7]. The symptom cluster that defines the metabolic syndrome has been shown to contribute to the pathogenesis and progression of type 2 diabetes and its related disorders, cardiovascular disease (CVD), and other chronic conditions [4, 6–14]. 

Other abnormalities associated with the metabolic syndrome and linked to the pathogenesis and progression of chronic diseases include hypercoagulation, chronic inflammation, endothelial dysfunction, oxidative stress, and reduced bioavailability of insulin-like growth factor-1 [8, 15–17]. Increased sympathetic activity and reduced parasympathetic tone have been implicated in the pathogenesis of the metabolic syndrome and its related complications [15, 18]. In addition, there is mounting evidence that chronic psychological stress and negative mood states are strongly associated, in a bidirectional manner, with insulin resistance, glucose intolerance, central obesity, dyslipidemia, hypertension, and other components of the metabolic syndrome [19–25]. Likewise, prospective studies have shown that depression, a common comorbidity in individuals with the metabolic syndrome, increases subsequent risk for developing type 2 diabetes by two- to threefold [21, 26]. Sleep disturbance, which also increases with age [27] and has been implicated in the development of insulin resistance [28], has also been associated with an increased risk for both type 2 diabetes [29] and its associated complications, as well as other chronic conditions [30]. Given the dramatically increasing prevalence, associated premature mortality, disability, complications, and social and economic costs, management of the metabolic syndrome is of importance to public health.

Modalities such as yoga, tai chi, qigong, and meditation may represent adjuncts to the conventional care and management of the metabolic syndrome. Records of these practices extend back to ancient times in Asia, and many of these forms, now referred to as mind-body therapies, currently are practiced throughout the world. Use of mind-body practices have increased in the US in recent years. Around 20% of the population engage in some form of mind-body practice based on data from the 2007 National Health Interview Survey, with yoga, meditation, and deep breathing exercises being the most popular [31]. Mind-body modalities have demonstrated efficacy in improving individual indices that comprise the symptom cluster that defines the metabolic syndrome. A recent comprehensive review found that yoga improved specific metabolic risk factors, including blood pressure, lipoprotein profiles, body mass index (BMI), and insulin sensitivity [32]. Clinical trials have demonstrated the benefits of yoga in diabetes, hypertension, dyslipidemia, atherosclerosis, improving blood glucose [33, 34], the insulin-glucose ratio, glycosylated hemoglobin [34], and requirements for oral hypoglycemics and insulin [33] as well as body weight and composition [32].

Growing evidence suggests that tai chi and qigong may improve indices of glycemic control in people with diabetes [35]. Qigong has been shown to reduce blood pressure [36], insulin resistance, glucose intolerance, oxidative stress [37], and other related indices of CVD risk [35, 36]. While research is limited, findings suggest the use of qigong programs for reducing stress, anxiety, and depression and improving associated health outcomes, including promotion of adequate sleep [38]. Randomized, controlled trials (RCTs) of transcendental meditation, a modality restored from ancient Vedic tradition in India and taught worldwide since 1957, have demonstrated blood pressure-lowering effects similar to primary antihypertensive medications [39]. 

Although the mechanisms underlying the putative physiologic and psychological effects of the practice of mind-body modalities are not yet well understood, the observed changes likely occur through a number of pathways. By reducing the activation and reactivity of the sympathoadrenal system and the hypothalamic pituitary adrenal (HPA) axis and promoting feelings of well-being, mind-body therapies may alleviate the effects of stress and foster multiple positive downstream effects on neuroendocrine status, metabolic function, and related inflammatory responses. Also, by directly activating the vagus nerve, mind-body practices may enhance parasympathetic output and thereby shift the autonomic nervous system balance from primarily sympathetic to parasympathetic, leading to positive changes in cardiac-vagal function, mood, energy state, and related neuroendocrine, metabolic, and inflammatory responses [40]. The demands of modern society may be responsible for higher levels of chronic stress, leading to activation of the neurohormonal system, specifically the sympathoadrenal system and HPA axis that involves catecholamine release, vagal withdrawal, cortisol secretion, and upregulation of the renin-angiotensin system. While research remains limited, findings suggest that the use of mind-body practices reduces stress, anxiety, and depression and improves associated health outcomes. These benefits may be particularly important for people at risk of or with the metabolic syndrome, who may be more vulnerable to compromised health-related quality of life, poor psychosocial health, poor self-care practices, increased healthcare costs, and adverse outcomes [41].

This systematic review critically evaluates the data examining the efficacy of mind-body therapies as supportive care modalities for management of the metabolic syndrome, focusing on studies of the cluster of symptoms that make up the metabolic syndrome rather than on those studies that have investigated only single components. 

## 2. Methods

### 2.1. Data Sources

Electronic databases (MEDLINE, CINAHL, and PubMed) were searched from the respective inception dates through November 18, 2010, to locate potentially relevant peer-reviewed articles using logical combinations of the following search terms: “mind-body,” “metabolic syndrome,” “yoga,” “tai chi,” “qigong,” “mindfulness,” and “meditation”. Additionally, relevant journals and the references of all located articles were manually searched for other potentially relevant studies. 

### 2.2. Study Selections

For the purposes of this systematic review, mind-body therapies were limited to yoga, tai chi, qigong, mindfulness-based techniques, and any form of meditation. Studies that assessed one of the defined mind-body therapies alone or as an adjuvant to conventional treatment in human subjects with the metabolic syndrome were included. Trials were excluded if the study examined a mind-body modality as part of a complex intervention (i.e., combining a mind-body therapy with another complementary modality), was aimed at the development of the methodology of mind-body therapy procedures without clinical outcomes, reported no data or statistical comparisons, assessed healthy subjects, or was limited to only components of the metabolic syndrome (i.e., hypertension or insulin sensitivity alone). Abstracts were included, and hard copies of all documents pertaining to the included studies were obtained and read in full. 

### 2.3. Data Extraction and Quality Assessment

Two independent reviewers validated, extracted, and recorded relevant study data using predefined criteria. Allocation concealment was assessed using the Cochrane classification, and the quality of all studies was independently assessed using a modification of the scoring criteria used for a previous review of a biofield therapy by the authors [44]. Using this method, a maximum of five points was awarded. One point was given for a description of the trial as a randomized study, one point for an appropriate method of randomization being reported, one point for an appropriate control group, and one point for the blinding of the evaluator. Given the nature of the interventions, blinding of the study participants is not possible and was not included in the scoring of the trials. An additional point was awarded for reporting the details concerning withdrawals and dropouts from the trial. Any discrepancies in the scoring of the trials were resolved by discussion between the two reviewers. 

## 3. Results

### 3.1. Study Description

**Figure 1:**
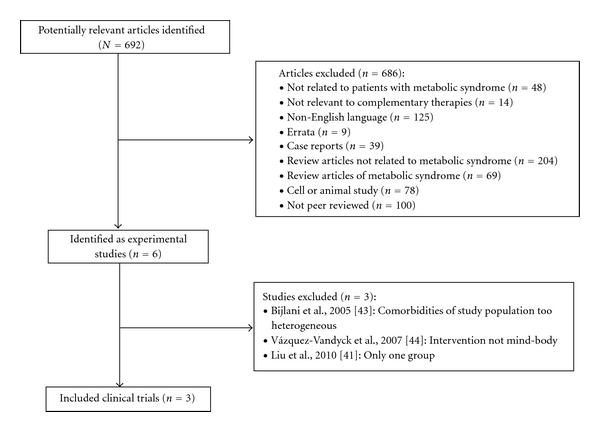
Diagram of review process and trial selection.

The searches identified 692 potentially relevant articles, of which 689 articles were excluded. This resulted in three trials being included in the paper. A schematic of the excluded studies as well as the reasons for exclusion are outlined in Figure 1, with key data from each study summarized in Table 1. One study was excluded because the comorbidities of the study population were too heterogeneous and not limited to the metabolic syndrome [45]. Another study was excluded because the intervention did not have a strong mind-body component and was related more to physical activity alone than to a mind-body modality [46]. An additional study was excluded because it was of a single-group, quasiexperimental design [41]. 

### 3.2. Study Quality

The quality scores of the included RCTs ranged between 2 and 5 out of possible 5 points. Of the three included trials, only one adequately described the methods of randomization and reported sufficient information regarding appropriate allocation concealment and assessor blinding [39]. Details of study dropouts and withdrawals were reported in two trials [39, 43]. 

### 3.3. Included Studies

Paul-Labrador and colleagues [39] (modified score = 5) conducted an RCT of transcendental meditation using a two-group, parallel design in individuals with coronary heart disease and the metabolic syndrome. Subjects (*N* = 103) were randomized to either a transcendental meditation or a health education program for 16 weeks. Both the meditation and health education interventions consisted of the same number and frequency of group meetings. The transcendental meditation intervention involved a highly standardized, traditional protocol, while the health education intervention was comprised of lectures and discussions involving risk factors for and the impact of lifestyle and environmental factors on CVD. Outcome measures included blood pressure, lipoprotein profile, insulin resistance using the homeostasis model assessment (HOMA), endothelial function using the brachial artery reactivity test (BART), heart rate variability (HRV), medical history, body mass index (BMI), hostility, depression, trait anxiety, trait anger, stress, plasma cholesterol, triglycerides, HDL cholesterol, plasma glucose, plasma insulin, and high-sensitivity C-reactive protein (*hs*-CRP). 

A significant difference in adjusted systolic blood pressure was observed at the end of the study in the transcendental meditation group versus the health education group (*P* = .04). No differences were found in lipoproteins, *hs*-CRP, or BMI. There was a trend toward an increase in physical activity in the health education group versus the transcendental meditation group. The health education group was more depressed and angry at the beginning and end of the study period than was the transcendental meditation group. Improved plasma glucose and insulin levels were observed in the transcendental meditation group versus the health education group at the conclusion of the study period (*P* = .01). No difference in endothelial function was observed. A significant difference in the change in the high-frequency HRV was observed in the transcendental meditation group versus the health education group at the end of the study after adjusting for covariates (*P* = .02), with a trend toward a change in the measurements of total power and low-frequency HRV (*P* = .07). 

Khatri and colleagues [42] (modified score = 2) evaluated the efficacy of a yoga intervention plus usual care versus usual care alone in individuals (*N* = 101) with the metabolic syndrome in a randomized study over the course of 12 weeks. Outcome measures included BMI, waist circumference, blood pressure, blood glucose, glycosylated hemoglobin (HbA1c), triglycerides, and HDL. Significant improvements in waist circumference, systolic and diastolic blood pressure, blood glucose, HbA1c, triglycerides, and HDL (*P* < .001) were observed in the yoga group from baseline to the end of the study period. A comparison to the control group was not given.

Cohen and colleagues [43] (modified score = 3) examined the effects of a restorative yoga intervention using a parallel study design. The yoga intervention consisted of a 3-hour introduction class, followed by a 90-minute class two times per week for 5 weeks, and then one class weekly for 5 weeks. Subjects (*n* = 24) randomized to the yoga group were to practice 3 times per week outside of class, tracking activity in a log book. The control group received no intervention. Outcome measures included BMI, waist circumference, blood pressure, insulin sensitivity, plasma glucose, plasma insulin, triglycerides, HDL, LDL, demographic information, perceived stress (PSS), dietary information using a food frequency questionnaire (2005 Block questionnaire), physical activity, quality of life (SF-36), depression (CES-D), and a self-rating of overall health using a Likert scale. Trends toward improved blood pressure (*P* = .07), well-being (*P* < .12), and stress (*P* < .22) were observed, as well as nonsignificant changes in weight and BMI in the yoga group. There was no difference in any of the serological measures in either group at the end of the study. The yoga group reported a significant improvement in energy level (*P* < .009). No significant changes in diet or physical activity were reported. The yoga group reported being “very satisfied” with the intervention, with the majority (87%) finding the practice of restorative yoga “very easy” or “moderately easy.” This study demonstrates the feasibility of recruiting overweight individuals with the metabolic syndrome for a yoga study. 

## 4. Discussion

A very small number of clinical trials addressing the use of mind-body therapies for management of the metabolic syndrome were identified in the process of conducting this review. Though the studies support the potential clinical effectiveness of mind-body practices in improving indices of the metabolic syndrome, more studies are required given that the trials included had several limitations. In the study by Paul-Labrador and colleagues [39], a lack of a description of the psychosocial outcome measures used as well as baseline differences in depression and anger make the results difficult to interpret. Moreover, a usual care alone group was not included in the study, which is essential when making comparisons to the standard of care. The use of HOMA as a measure of insulin resistance is an indirect method that may have underestimated effects of the interventions in the reviewed studies. None of the studies indicated the use of ambulatory blood pressure monitoring over the course of the study. Measurement of blood pressure only at study intervals might have underestimated the efficacy of the mind-body interventions in lowering this key component/symptom of the metabolic syndrome. Only one of the reviewed studies did not include waist circumference as an outcome measure [39]. The study by Khatri and colleagues [42] was published as a brief correspondence, limiting the description of the study population, the methods, the intervention, and the results, including a comparison of the intervention with the control group. No rationale was given for the inconsistency in the duration and timing of the intervention over the course of the study period in the trial by Cohen and colleagues [43]. Additionally, this latter study had a small sample size limiting the statistical power of the results, a brief follow-up period that may have diminished the estimation of effects, and those participants in the yoga group may have been affected by the group dynamics of the yoga classes. 

For the results of a clinical study to be useful, one must be able to replicate the trial; therefore, all aspects of the methodology and the intervention, as well as a detailed description of the results, must be reported. None of the included studies in this paper provided a clear rationale for the treatment specificity or duration. Given that the optimal dosage of mind-body therapies has yet to be determined, a description of the treatment duration and number of treatments should be included. While there remains a lack of rigorous trials that apply adequate methodology, including the use of blinding and placebo treatments, given that trials with inadequate levels of blinding are likely to show exaggerated treatment effects [47], the nature of mind-body therapies makes it seemingly impossible to blind subjects to the intervention or to develop a placebo.

Given the physiological outcomes associated with the metabolic syndrome, objective outcome measures such as lipoprotein profiles, circulating levels of glucose and insulin, and anthropometric measures are consistent across studies of the metabolic syndrome, which allows for comparison among studies. However, different subjective assessments were used in the included studies to determine subjective or psychosocial outcomes such as fatigue and health-related quality of life, making comparisons more challenging. This issue is not unique to the study of mind-body or complementary therapies. Indeed, RCTs often use various outcome measures of patient symptoms to quantify the same concepts, limiting comparison across studies [48]. Additionally, some patient-reported outcomes are likely not to be captured because of a lack of sensitivity of the instrument or to the floor and ceiling effects of some measures that do not assess symptom extremes adequately [48], a potential limitation acknowledged by Paul-Labrador and colleagues [39]. The Patient-Reported Outcomes Measurement Information System (PROMIS) Initiative aims to improve appreciably how these measures are selected and assessed in clinical research, including clinical trials [48]. As part of the NIH Roadmap Initiative, use of these measures supports the goals of the PROMIS Initiative to revolutionize the way patient-reported outcome tools are used in clinical research and practice and to aid in transforming the nation's medical research capabilities, speeding the movement of research [49].

Limitations of systematic reviews, including the current paper, relate to any potential incompleteness of the reviewed studies. This effect may result from publication bias given that negative studies tend to remain unpublished [50]. A further weakness of the current paper involves the limitations of the reviewed studies. Methodological shortcomings such as small sample size and inadequate reporting of methods and results leave the overall result inconclusive. The exclusion of studies focusing on clinical outcomes related to single components of the metabolic syndrome may be a limitation; however, the inclusion of trials examining only certain cardiometabolic measures would not provide reliable data on the clinical effectiveness of mind-body therapies in improving the symptom cluster that comprises the metabolic syndrome as a whole.

Mind-body therapies may carry practical advantages as therapeutic interventions for managing the symptom cluster associated with the metabolic syndrome. While several large trials have demonstrated the effectiveness of physical activity in improving outcomes of the metabolic syndrome, many individuals, such as those who are overweight and sedentary and at highest risk for the metabolic syndrome, may be unable or unwilling to participate in conventional types of physical activity (e.g., strength training and gym-based exercises) [41, 43]. Mind-body modalities are simple, economical, noninvasive therapies, easy to learn, and can be practiced easily by individuals who may experience potential limitations in mobility [51]. Requiring little in the way of equipment or professional personnel, mind-body therapies are relatively easy and inexpensive to practice. Moreover, mind-body therapies typically bring immediate positive benefits, including feelings of relaxation and tranquility. For example, even short-term qigong programs (20 days to 10 weeks) have been shown to result in significant improvements in sleep, mood, and other distressful symptoms, as well as cardiovascular health [35, 52], helping to encourage continued practice. Several studies indicate that qigong and other mind-body programs may reduce healthcare costs in both clinical and nonclinical populations [53, 54]. 

## 5. Conclusion

Clearly, there is a need to identify cost-effective prevention and management strategies for the metabolic syndrome that address the multiple interrelated factors underlying this complex, devastating, and rapidly increasing chronic condition. No such research has been conducted in this regard with respect to mind-body therapies. Current clinical practice guidelines indicate lifestyle modifications as the sole therapy for prehypertension, as well as other indicators of the metabolic syndrome [55]. Given the positive effects of mind-body therapies on cardiometabolic components, these modalities most likely would be of benefit to individuals with the metabolic syndrome. The current paper provides healthcare practitioners with information that could be used in decision-making about recommendations involving mind-body practices. In light of the important role of psychosocial factors in the development of insulin resistance, type 2 diabetes, and other chronic diseases, the influence of sympathetic activation in the pathogenesis of insulin resistant states and the bidirectional relationships of these and other insulin resistance-related risk factors and mind-body therapies may hold promise for both the prevention and treatment of the metabolic syndrome. Because RCTs remain the “gold standard” in biomedical research, this paper highlights the need for such trials of mind-body therapies with regard to the management of the metabolic syndrome, given the relative absence of such studies in the literature, as well as the mechanisms of action involved in mind-body therapies. 

## Figures and Tables

**Table 1:** Summary of clinical studies of mind-body therapies.

References	Design, allocation concealment*	Modified score	Mean age, sample size (randomized/analyzed), condition other than metabolic syndrome	Interventions (regimen)	Main outcome measures	Main results	Comments
Paul-Labrador et al., 2006 [39]	Parallel 2 groups Unmasked	5	Mean age = 67.4 Sample size = 103 Coronary heart disease	Transcendental meditation versus health education for 16 weeks	Blood pressure, lipoprotein profile, insulin resistance (HOMA), endothelial function (BART), HRV, medication history, BMI, hostility, depression, trait anxiety, trait anger, stress, plasma cholesterol, triglycerides, HDL-C, plasma glucose, plasma insulin, and *hs*-CRP	Significant difference in systolic and arterial blood pressure at exit No difference in lipoproteins, *hs*-CRP, BMI, or BART Improved glucose, insulin levels, and HRV in meditation group	Trend toward increase in physical activity in health education group Health education group more depressed and angry at entry and exit

Khatri et al., 2007 [42]	Parallel 2 groups Unknown	2	Mean age = 54.01 Sample size = 101	Usual care versus usual care plus yoga for 12 weeks	BMI, waist circumference, blood pressure, blood glucose, HbA1c, triglycerides, HDL-C	Significant improvement in waist circumference, blood pressure, blood glucose, HbA1c, triglycerides, and HDL-C in yoga group	

Cohen et al., 2008 [43]	Parallel 2 groups Unknown	3	Mean age = 52 Sample size = 24	Restorative yoga versus control	BMI, waist circumference, blood pressure, insulin sensitivity, plasma glucose, plasma insulin, triglycerides, HDL-C, LDL-C, demographics, PSS, Block 2005, physical activity, SF-36, CES-D, overall health (Likert scale)	Trend toward improved blood pressure, well-being, and stress in yoga group Nonsignificant changes in BMI in yoga group No difference in serological measures, diet, or physical activity	Yoga group reported being “very satisfied” with intervention, felt that practicing restorative yoga “very easy” or “moderately easy”

*Classified by Cochrane criteria. HOMA: homeostasis model assessment; BART: brachial artery reactivity test; HRV: heart rate variability; BMI: body mass index; HDL-C: high-density lipoprotein-cholesterol; *hs*-CRP: high-sensitivity C-reactive protein; HbA1c: hemoglobin A1c; LDL-C: low-density lipoprotein cholesterol; PSS: Perceived Stress Scale; Block 2005: food frequency questionnaire; CES-D: Center for Epidemiologic Study-Depression Scale; PSQ: Perceived Stress Questionnaire; SF-36 (measures physical and mental aspects of health).
